# Understanding patterns of adherence to COVID-19 mitigation measures: a qualitative interview study

**DOI:** 10.1093/pubmed/fdab005

**Published:** 2021-02-09

**Authors:** Sarah Denford, Kate S Morton, Helen Lambert, Juan Zhang, Louise E Smith, G James Rubin, Shenghan Cai, Tingting Zhang, Charlotte Robin, Gemma Lasseter, Mathew Hickman, Isabel Oliver, Lucy Yardley

**Affiliations:** Population Health Sciences, Bristol Medical School, University of Bristol, Bristol BS8 1UD, UK; School of Psychological Science, University of Bristol, Bristol BS8 1TU, UK; NIHR Health Protection Research Unit (HPRU) in Behavioural Science and Evaluation, University of Bristol in collaboration with Public Health, UK; Academic Unit of Psychology, University of Southampton, Southampton SO17 1BJ, UK; Population Health Sciences, Bristol Medical School, University of Bristol, Bristol BS8 1UD, UK; NIHR Health Protection Research Unit (HPRU) in Behavioural Science and Evaluation, University of Bristol in collaboration with Public Health, UK; Population Health Sciences, Bristol Medical School, University of Bristol, Bristol BS8 1UD, UK; NIHR Health Protection Research Unit in Emergency Preparedness and Response, King’s College London, London SE5 9RJ, UK; Department of Psychological Medicine, King’s College London, London WC2R 2LS, UK; NIHR Health Protection Research Unit in Emergency Preparedness and Response, King’s College London, London SE5 9RJ, UK; Department of Psychological Medicine, King’s College London, London WC2R 2LS, UK; Population Health Sciences, Bristol Medical School, University of Bristol, Bristol BS8 1UD, UK; Population Health Sciences, Bristol Medical School, University of Bristol, Bristol BS8 1UD, UK; NIHR Health Protection Research Unit (HPRU) in Behavioural Science and Evaluation, University of Bristol in collaboration with Public Health, UK; Public Health England, National Infection Service, Liverpool, UK; NIHR Health Protection Research Unit in Emerging and Zoonotic Infections, University of Liverpool, Liverpool L69 7BE, UK; NIHR Health Protection Research Unit in Gastrointestinal Infections, University of Liverpool, Liverpool L69 3BX, UK; Population Health Sciences, Bristol Medical School, University of Bristol, Bristol BS8 1UD, UK; NIHR Health Protection Research Unit (HPRU) in Behavioural Science and Evaluation, University of Bristol in collaboration with Public Health, UK; Population Health Sciences, Bristol Medical School, University of Bristol, Bristol BS8 1UD, UK; NIHR Health Protection Research Unit (HPRU) in Behavioural Science and Evaluation, University of Bristol in collaboration with Public Health, UK; Public Health England, National Infection Service, Bristol BS1 6EH, UK; Population Health Sciences, Bristol Medical School, University of Bristol, Bristol BS8 1UD, UK; School of Psychological Science, University of Bristol, Bristol BS8 1TU, UK; NIHR Health Protection Research Unit (HPRU) in Behavioural Science and Evaluation, University of Bristol in collaboration with Public Health, UK; Academic Unit of Psychology, University of Southampton, Southampton SO17 1BJ, UK

**Keywords:** adherence, COVID-19, infection control, public involvement, qualitative, risk assessment

## Abstract

**Background:**

Evidence highlights the disproportionate impact of measures that have been introduced to reduce the spread of coronavirus on individuals from Black, Asian and minority ethnic (BAME) communities, and among those on a low income. An understanding of barriers to adherence in these populations is needed. In this qualitative study, we examined the patterns of adherence to mitigation measures and reasons underpinning these behaviors.

**Methods:**

Semi-structured interviews were conducted with 20 participants from BAME and low-income White backgrounds. The topic guide was designed to explore how individuals are adhering to social distancing and self-isolation during the pandemic and to explore the reasons underpinning this behavior.

**Results:**

We identified three categories of adherence to lockdown measures: (i) caution-motivated super-adherence (ii) risk-adapted partial-adherence and (iii) necessity-driven partial-adherence. Decisions about adherence considered potential for exposure to the virus, ability to reduce risk through use of protective measures and perceived importance of/need for the behavior.

**Conclusions:**

This research highlights a need for a more nuanced understanding of adherence to lockdown measures. Provision of practical and financial support could reduce the number of people who have to engage in necessity-driven partial-adherence. More evidence is required on population level risks of people adopting risk-adapted partial-adherence.

## Introduction

Numerous mitigation measures have been introduced in an effort to prevent the spread of coronavirus (COVID-19) in the UK. These include restrictions of movement, social distancing, mandatory use of face coverings in certain settings and engagement in test, trace and isolate procedures when necessary. Critically, the effectiveness of these measures for preventing transmission is dependent on the extent to which they are known, understood, accepted and adopted in time. However, throughout the pandemic, research indicates variation in the extent to which people are willing and able to adhere to guidance.^1–4^

Although social distancing and self-isolation behavior during lockdown can be crudely defined as being ‘adherent’ or ‘not-adherent’, it is likely that there is a more nuanced scale of adherence.^5^ Attempts have been made to challenge the view that adherence should be considered a dichotomy. Fancourt *et al.*^6^ use the terms ‘complete’ and ‘majority’ adherence to compare those who follow all the guidance all the time with those who follow some of the guidance, or for some of the time. Williams *et al.*^5^ refer to ‘overt rule breaking’ and ‘subjective rule interpretation’ to differentiate between those who were deliberately breaking the rules, and those who are interpreting inconsistent or constantly changing guidance to suit their needs. However, these terms do not capture the complexities underpinning decisions to adhere to the guidance, and the risk that this may bring. People may be acting conscientiously, engaging only in activities in which they are unlikely to come into contact with anyone, attempting to minimize the risk of transmitting or catching the virus.^5^ Alternatively, people may be leaving their home out of necessity, in order to buy food or medicine, or to provide care for a vulnerable person. It is still unclear how the public are making decisions about what they should and should not do, what they actually are doing, and how safe this is.

Periods of lockdown may be particularly challenging for those from the lowest income backgrounds and individuals from Black, Asian and minority ethnic (BAME) communities who are less able to engage in social distancing measures, and are less able to work from home and self-isolate when required.^2^ Emerging evidence from the Mental Health Foundation’s Mental Health in the Pandemic study also indicates that a higher proportion of members of BAME communities are experiencing financial concerns, fear and anxiety than members of the non-BAME population. Furthermore, people from BAME communities are more likely to be in precarious work and where furloughing may not have been offered.^7^ Understanding and supporting adherence to mitigation measures among this population is therefore critical.

The aim of this study was to gain a better understanding of how people from low-income and BAME communities are adhering to social distancing and self-isolation measures during the COVID-19 pandemic, and to explore in detail the reasons underpinning this behavior.

## Methods

Participants over the age of 18 years from BAME, and low-income White backgrounds were recruited via social media channels. We invited interested individuals to contact the research team via email. Potential participants were sent an information sheet about the study. All interviews were conducted via the telephone or using the online platform Zoom. Audio-recorded verbal consent was obtained.

Interviews were conducted between the 8th and 31st July 2020 and lasted between 21 and 55 min. At that time, non-essential shops and places of worship were allowed to open, and in England, groups of six were allowed to meet outside. People were still required to stay 2 m apart, and isolate if they, or their household, experienced symptoms of COVID-19. Face masks were compulsory on public transport, and from the 24th July were also mandatory in shops. Participants were asked about their understanding and perceptions of government mitigation measures, and their decisions about social distancing and self-isolation behavior during lockdown.

Following the stages of thematic analysis,^8^ two researchers independently read transcripts and assigned initial codes to the data. Possible themes were identified and refined through discussion. Data were checked against an initial framework and refinements made as necessary. For each theme in the framework, charts were developed, and relevant text copied verbatim. Charts were used to identify common concepts within and between participants, and explanations sought for divergence. Participants were given the opportunity to discuss the analysis and interpretations with the researcher team via Zoom or telephone meetings. Two participants engaged in these discussions, contemplating how their behavior fit with the identified categories of adherence, and providing feedback on the final themes.

## Results

A total of 20 participants (13 female) took part in the interviews. Participants were between the ages of 18 and 65 years and from Black African and Black Caribbean (*N* = 4), Asian^9^ and White (*N* = 7) ethnic groups. The average (mean) Index of Multiple Deprivation decile was 4.15. Four participants reported that they had had COVID-19, or symptoms of COVID-19 in the household.

## Results of the thematic analysis

**Fig. 1:**
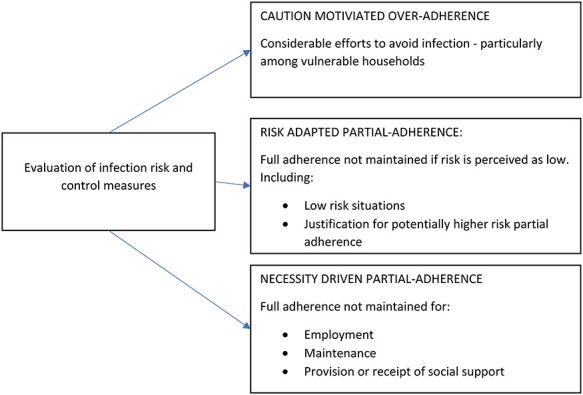
Patterns of adherence to social distancing and self-isolation measures during the COVID-19 pandemic.

Thematic analysis showed that participants engaged in active evaluation of infection risk and control measures, following which three context-specific patterns of adherence were identified (Fig. 1).

### Evaluation of infection risk and control measures

Participants were eager to understand how they could protect themselves, their households and their communities, but often struggled to keep up-to-date with and make sense of the ‘constantly changing’ advice and recommendations (Table 1—Quote 1). There was evidence of a lack of understanding of terms such as self-isolation, and how to self-isolate safely (Quote 2). Participants were unclear as to whether they should be isolating (Quote 3), or shielding (Quote 4), and felt uniformed about the number of cases of COVID-19 in their local areas (Quote 5).

**Table 1:** Evaluation of infection risk and control measures

Quote 1	‘I think the speed at which things were changing was quite confusing. One day there would be one set of principles and then the next day it was all completely changed. It was hard to keep up’ (Female, White)
Quote 2	‘I always found that quite confusing about who should isolate. The guidance around them, and how many days’ (Participant 20, White Male).
Quote 3	‘Um there were a couple of times we may have been displaying symptoms, but we knew, we had not isolated for one of those times in fact, to be honest. I guess we took, I would say a vaguely educated risk because no one knew what was happening really’ (Participant 03, BAME Male)
Quote 4	‘I’ve got high blood pressure and so then that… in some places, in some of the reports it put me at risk. Then some it did not’ (Participant 01, BAME Female).
Quote 5	‘You want to understand how many people are affected in ((region)). How many deaths. And so on, and so forth. So, you can start to form your opinions’ (Participant 08, BAME Male).
Quote 6	‘I think people could interpret it the way that they wanted to’ (Participant 14, White Female).
Quotes 7 and 8	‘You cannot trust everything a government says… we have to use our common sense and other things to survive’ (Participant 11, BAME Female). ‘It’s down to me to decide how I interpret information and take it on board’ (Participant 03, BAME Male)
Quote 9	‘You’ve got to look after yourself and your family and do with is right for yourself and your family and you will not go far wrong’ (Participant 13, White Female).

Due to a lack of scientific certainty surrounding the guidance, messages were often considered to be ‘open to interpretation’ (Quote 6). Participants described a need to decide for themselves how to respond to the information they were receiving, and to use their judgement, rather than simply following the guidance (Quotes 7 and 8). Decisions appeared to be made on the basis of their individual situations, putting the health and needs of their family ahead of government guidance (Quote 9).

### Caution-motivated super-adherence

Participants described a willingness to adhere to mitigation measures in order to protect themselves and their households from the virus (Table 2—Quote 1), or to protect others around them, for example, if and when they had experienced symptoms of COVID-19 (Quote 2). Increased efforts were taken by participants from vulnerable groups to protect themselves (Quote 3) or vulnerable members of their households. Many of the participants who considered themselves or their households to be vulnerable, and felt that the risk of exposure to the virus was high, reported engaging in additional, precautionary measures to protect themselves and their families (Quote 4). Super-adherence was particularly prominent among vulnerable participants who did not consider government recommendations to be sufficient for protecting them (Quote 5).

**Table 2:** Caution-motivated super adherence

Quote 1	‘I did obey because I do not want to get sick and drop dead’ (Participant 01, BAME Female)
Quote 2	‘But we do have to be selfless and just [adhere to the restrictions] for the rest of your community and the nation’ (Participant 02, BAME Female).
Quote 3	‘Now we are extremely careful here. Being of Asian origin myself I know there’s more chances of me catching COVID like Asian and African people than your—compared to Caucasian people, so I’m being extra careful’ (Participant 12, BAME Male).
Quote 4	‘I think when the news came in around the death rate was increasing, I decided after two weeks or a week not to go shopping at all and avoid the supermarket’ (Participant 05, BAME Male).
Quote 5	‘I do not feel protected by the government. I felt the easing of the lockdown felt a bit early. I carried on exactly the same for a while’ (Participant 15, White Female).

### Risk-adapted partial-adherence

A second pattern of adherence included breaking lockdown rules if it was perceived as safe to do so (Table 3). Partially-adherent behaviors were justified by participants, either because they were genuinely perceived as being low risk or because there were sufficient inconsistencies in key messages to allow participants to present their behavior as low risk. Low risk behaviors included those that were considered unlikely to contribute to the transmission of COVID-19 (e.g. those that do not result in close contact with others), as well as behaviors that were considered safe for the individual participant. As an example of the former, one participant described a willingness to leave the house on more than one occasion, a behavior that was not permitted at the time, because he considered it very unlikely that he would come into contact with others (Quote 1). Behaviors that were considered safe for the individual were usually based on individual perceptions of risk, with those who did not consider themselves to be at high risk describing how this had made them less inclined to adhere to social distancing guidelines (Quotes 2 and 3), whereas potentially overlooking the risk to others.

**Table 3:** Risk adapted partial adherence

Quote 1	‘But the fact that my exercise took place between sort of five and six in the morning, it felt kind of almost like that did not count where I’d see one person maximum. And I think well I’m not really interacting with anyone there, so I get a free pass’ (Participant 20, White Male)
Quotes 2 and 3	‘[I was] not so worried about catching it. I guess there are levels of how well I’d be able to deal with it, but I would not say that I was being incredibly precautionary. I was taking the basis measures… I wasn’t incredibly cautious with the measures I was taking’ (Participant 03, BAME Male). ‘I mean, it’s a bit selfish, but we know that we as a family do not come into the category of, you know, highly vulnerable people’ (Participant 06, BAME Female).
Quotes 4 and 5	‘Even on the government website say that it’s no longer very – how to say it—dangerous disease that you know can kill everyone. Based on the night we saw that—and even BBC said it’s not that critical’ (Participant 13, BAME Female). ‘I think, yeah, to be fair the infection rates—we are being told—are coming down, I think perhaps. Human nature being what it is, I think we are all a little bit less watchful’ (Participant 14, White Male).
Quote 6	‘[Neighborhood children] have been playing with each other, but again rigorous hand washing when he comes in, when my son comes in I say wash your hands, wash your face...You have to get out, you have to go out at some point, and the fact that there is a lot, some of the children were allowed in school, year groups, so I let him’ (Participant 17, White Female)
Quote 7	‘I felt because I was wearing a mask, gloves and not going near anyone, it wasn’t much different to going out in the country and see people at a distance, so I do not know. I did that with other people that had a need, with two friends’ (Participant 16, White Female).
Quote 8	‘Then obviously sort of the media got onto things about sort of famous people who had, well not famous people but people in power who had bent the rules to suit their needs and things like that and then it starts to sort of—and then you see people just disregarding it and you kind of think, well why am I bothering to sort of restrict my lifestyle to suit when there’s certain people who aren’t bothered’ (Participant 20, White Male)

Any apparent ambiguities or inconsistencies in information could be used to justify partial-adherence (Quotes 4 and 5). One participant explained how she had allowed her son to play with his friends as key worker children were allowed to remain in school (Quote 6). Another participant was willing to meet a friend during lockdown because she reasoned that it was no different from seeing others’ outside at a distance (Quote 7). A third participant, who described himself as low risk, outlined how breaches of lockdown among influential figures reduced his willingness to restrict his own lifestyle (Quote 8).

### Necessity-driven partial-adherence

Participants described situations in which they felt they had no choice but to break the rules around social distancing or self-isolation. Participants described a need to find a balance between staying safe and maintaining their mental health and wellbeing (Table 4: Quote 1), or to continue to work because of financial concerns and responsibilities (Quote 2). Indeed, there was perceived pressure from those in management positions to return to work—even when it was against official advice (Quote 3). One participant who lived in rented accommodation felt under pressure to allow her landlord to enter her home for maintenance purposes, even though she did not feel comfortable allowing him to do so (Quote 4). Other necessities included for religious purposes (Quotes 5 and 6), or to provide support for bereavement (Quote 7).

**Table 4:** Necessity-driven partial-adherence

Quote 1	‘Initially there was a lot of worry, it’s still obviously taking precautions, but now it’s kind of trying to find the balance between making sure you are sticking to the guidelines and being extra safe while still trying to maintain your wellbeing and social interaction, and that side of things’ (Participant 10, BAME Female).
Quote 2	‘They might have just a cold or a sniffle and they would come in [to work]. I know that because they are not on a big wage, and I know you can get the sickness and all the rest of it, but it’s not going to help when you have got a mortgage to pay’ (Participant 18, White Female).
Quote 3	‘I had to narrate to my manager when he was trying to say ‘Why are you not coming in?’... it was into lockdown, April or May, and I was coming out of my illness... I’ve got the emails to prove it... I had to narrate. I had to narrate the government guidelines to stay at home’ (Participant 04, BAME Female).
Quote 4	‘Even if he was not allowed to come in because everyone was not – it was during the first month of the lockdown. No-one was allowed to go [out of the] home except for food and exercise. He came twice to have a look at the house. I do not know’ (Participant 07, BAME Female).
Quotes 5 and 6	‘I mean he [participant’s son] went to church because... well he felt he had to’ (Participant 08, BAME Female). ‘She’s got her church friends that goes round. But I think they just sit in the garden or sit in the kitchen, the two of them and do their bible studies’ (Participant 08, BAME Female).
Quote 7	‘I have been to see my step-mum, obviously, after the loss of my father’ (Participant 17, White Female).
Quote 8	‘I did actually realise I was in a bit of a state. I did go and have a distance walk with one of my sons and just basically talked’ (Participant 16, White Female)
Quote 9	‘I did then meet up with another friend who was finding lockdown difficult, which we were not supposed to at that point. We met at a distance on a walk. I felt mentally I needed that’ (Participant 16, White Female).
Quote 10	‘They’ve gone out for the last two months. They started going out to do exercises and go and meet up with friends’ (Participant 04, BAME Female).

A common motive for breaking social distancing guidance was for the sake of their own mental health and wellbeing, or that of their friends and family. One participant described how she had met with her son during lockdown as she was struggling with her anxiety (Quote 8). This participant later described going out during the lockdown period to meet a friend who was also struggling to cope with social distancing measures. Social contact with anyone outside the household was prohibited at the time (Quote 9). One parent was particularly anxious about the mental wellbeing of her children, and described how she had allowed her children to meet with friends before lockdown restrictions were lifted because she felt that they had needed it (Quote 10).

## Discussion

### Main findings of this study

Growing evidence highlights the substantial impact of the lockdown measures on individuals from BAME communities and those on low-income,^9^ with these individuals facing additional barriers to adherence to government imposed mitigation measures.^2^ In line with previous research, participants in the current study reported engaging in behaviors that were not always in line with the government’s social distancing and self-isolation advice. However, these acts of partial-adherence were not always high risk or avoidable. We suggest that participants made risk-adapted decisions based on their perceptions of the degree of transmission risk entailed by the behavior (for themselves and others), in relation to the importance of the activities they wanted or needed to undertake. We outline three context-specific patterns of adherence: (i) caution-motivated super-adherence, (ii) risk-adapted partial-adherence and (iii) necessity-driven partial-adherence. Our findings highlight the need for different forms of intervention, as well as additional research into the impact and risks associated with these patterns of adherence.

One pattern of adherence involved participants engaging in measures that were additional to those recommended by the government, and continuing to adhere to strict social distancing guidance after restrictions were lifted. Although participants in the current sample were able to continue with more stringent social distancing measures in the short term, it is critical that support is available for vulnerable individuals from BAME communities and those on low-income to be able to maintain this level of adherence when necessary. As much research highlights that social distancing is particularly problematic among these individuals,^2^ support is urgently required for vulnerable individuals who need to engage in additional protective measures when government-imposed restrictions are lifted.

A second pattern of adherence involved infringing rules around social distancing if it was perceived as safe to do so due to risk of transmission of COVID-19 being low (risk-adapted partial-adherence). Participants in the current study described leaving the home for physical activity on multiple occasions, or to meet with others at a safe distance outside the home (behaviors that were not permitted at the time). These behaviors were justified by participants as they were viewed as low risk; either because the participant would not be in close contact with others, or because alternative methods of protection (hand hygiene and face coverings) were used. Classifying this behavior as ‘risky’ in the same way as those who engage in high risk (e.g. indoor) contact may be unhelpful, particularly as physical activity is likely to have a positive impact on wellbeing.^10^^,^^11^ With essential social distancing measures in place this form of partial-adherence could potentially be a lower risk way of obtaining much needed social support.

In some situations, participants justified breaking social distancing rules because they did not consider themselves or their household to be vulnerable. The potential impact on transmission of COVID-19 beyond their household did not appear to have been considered. Other participants used comparisons to other situations in order to provide justification for their partial-adherence, such as key worker children being allowed to remain in school. However, the extent to which these behaviors or situations are genuinely low risk may be questioned. Indeed some participants, particularly those who considered themselves to be of low risk, appeared to be using information selectively to justify ignoring difficult social distancing rules. The cumulative impact of small acts of non-adherence is still unknown.^12^

A final pattern of adherence involved engaging in potentially risky behavior due to a perceived need or pressure (necessity-driven partial-adherence). This could be motivated by a need to maintain the mental health and wellbeing of themselves or others, to continue to work and earn a living, to deal with emergencies or for religious reasons. These participants perceived a critical need to engage in these behaviors and felt that they had no choice in the matter. These pressures are likely to be greater among individuals from BAME communities and those on a low-income, who are less able to work remotely or adhere to social distancing and self-isolation guidance.^7^^,^^9^ In order to improve engagement with lockdown measures, it is crucial that financial, tangible and social support is available.

### What is already known on this topic

Current understanding of adherence to social distancing and self-isolation has viewed adherence as a dichotomy, with the majority of research focusing on prediction rather than understanding adherence. Indeed, numerous surveys have identified influences such as age,^13^ gender,^13^ and ethnicity,^2^ perceptions of risk,^14^ behavior of others,^15^ access to help and support,^3^ trust in the government and the effectiveness of mitigation measures^15^^,^^16^ and already having had COVID-19^17^ on behavior. However, to date, research has not focused on exploring, in detail, what people are doing, why, and how safe it is.

### What this study adds

Our research has identified different patterns of adherence among BAME individuals and those on low income, each with different associated implications and risks. Although previous research depicts an overall lack of adherence to mitigation measures, we highlight that there are at least three patterns of adherence, and different forms of intervention will be needed to support individuals to protect themselves, their households and their communities from COVID-19 and the imposed mitigation measures. This may include provision of practical and social support for those who need it. Further research is needed to explore the impact and risks associated with categories of adherence, including the cumulative impact of small episodes of non-adherence at a population level. Although the individual breaking lockdown may consider themselves to be at low risk of the more serious consequences of COVID-19, the aim of the lockdown was to reduce contact between people to reduce the burden of disease overall in the population. Additional research is needed to understand the true impact of risk adapted partial-adherence on transmission of COVID-19.

### Limitations of this study

Although every effort was made to recruit a diverse and representative sample, we acknowledge that our use of social media may have resulted in a biased sample. Much of our recruitment was via COVID-19 support pages, and previous research has shown that use of social media during the pandemic is associated with increased levels of anxiety^18^ and misinformation.^19^ It is therefore possible that our samples of volunteers are not representative of those who do not use social media for COVID-19 related support or information. Likewise, participants did not necessarily have symptoms of COVID-19, and were therefore discussing breaches of social distancing, rather than self-isolation. Responses may have been different among a population who had experienced symptoms of COVID-19.

Our study may also have been influenced by response bias. It is possible that participants were unable to accurately recall attitudes and behaviors at the start of the pandemic, or did not feel able to disclose risky or substantial breaches of lockdown to the research team. Although participants in the sample were willing to share examples of partially-adherent behavior, they may not have been willing to share experiences of more risky behavior during the interviews.

Finally, interviews were conducted in July 2020. During this period, lockdown measures were being eased, and cases COVID-19 were falling. Alongside changing rules and guidance, knowledge, attitudes and behavior also change rapidly. Attempts to transfer the results of this study to other populations, or periods of lockdown must be made with caution.

## Conclusions

Although participants reported partially-adherent behavior, this was the result of a complex decision-making process regarding the risks and benefits of engaging in the behavior, often with clear attempts to reduce risk as much as possible. Participants appeared to actively make decisions to engage in behaviors that they considered to be safe and/or necessary, leading to three patterns of adherence. Our findings highlight the need for different forms of intervention, as well as additional research into the impact and risks associated with these patterns of adherence.

## Authors’ contributions

All authors conceptualized and designed the study and were involved in interpretation. SD, KM and LY were involved in data analysis. SD led the drafting of the manuscript. All authors reviewed the manuscript, approved the final content and met authorship criteria.

## Data Availability

The datasets used and/or analyzed during the current study are available from the corresponding author on reasonable request.

## Notes

**Sarah Denford**, Dr.

**Kate S. Morton**, Dr.

**Helen Lambert**, Professor

**Juan Zhang**, Dr.

**Louise E. Smith**, Dr.

**G. James Rubin**, Dr.

## Notes

**Shenghan Cai**, Dr.

**Tingting Zhang**, Dr.

**Charlotte Robin**, Dr.

**Gemma Lasseter**, Dr.

**Mathew Hickman**, Professor

**Isabel Oliver**, Professor

**Lucy Yardley**, Professor
